# Removal of muscular artifacts in EEG signals: a comparison of linear decomposition methods

**DOI:** 10.1007/s40708-017-0074-6

**Published:** 2018-01-10

**Authors:** Laura Frølich, Irene Dowding

**Affiliations:** 1 Technical University of Denmark, Lyngby, Denmark; 2 Technische Universität Berlin, Berlin, Germany

**Keywords:** Electroencephalogram (EEG), Artifact removal, Muscle artifacts, Blind source separation (BSS), Independent component analysis (ICA), Filtering

## Abstract

The most common approach to reduce muscle artifacts in electroencephalographic signals is to linearly decompose the signals in order to separate artifactual from neural sources, using one of several variants of independent component analysis (ICA). Here we compare three of the most commonly used ICA methods (extended Infomax, FastICA and TDSEP) with two other linear decomposition methods (Fourier-ICA and spatio-spectral decomposition) suitable for the extraction of oscillatory activity. We evaluate the methods’ ability to remove event-locked muscle artifacts while maintaining event-related desynchronization in data from 18 subjects who performed self-paced foot movements. We find that all five analyzed methods drastically reduce the muscle artifacts. For the three ICA methods, adequately high-pass filtering is very important. Compared to the effect of high-pass filtering, differences between the five analyzed methods were small, with extended Infomax performing best.

## Introduction

The removal of undesired artifacts from the electroencephalogram (EEG) is a major preprocessing step for most EEG analysis. Such artifacts stem from eye and muscle movement, the heart beat or external technical sources. In this paper, we are concerned with the removal of muscle artifacts. These are typically caused by muscle activity near the head, such as swallowing or head movements, and are characterized by high-frequency activity (> 20 Hz) [1]. Because muscle activity arises from different type of muscle groups, muscle artifacts are harder to stereotype than eye artifacts (cf. [2–4]).

The most widespread technique to reduce muscle artifacts in EEG recordings is the linear decomposition of EEG signals into source components. The overall goal is to separate artifactual from neural activity in different components, such that the artifactual components can be discarded and cleaner signals can be reconstructed from the neural components only. The most commonly deployed techniques to achieve this goal are variants of independent component analysis (ICA) [5–8], which solve the blind source separation (BSS) problem by maximizing the independence of the source components. In most cases, ICA methods yield a useful separation, but some mixed components remain which contain activity from both artifactual and neural origin (see, e.g., [9, 10] for a review of the current state-of-the-art).

While many different BSS/ICA methods are available, very few studies compared their performance on real EEG data. This is because, for a quantitative evaluation, the true presence or absence of artifacts in EEG signals (the ‘ground truth’) needs to be known or assumed on a sound basis. This is especially difficult for muscle artifacts, whose activity cannot be obtained from a single measurement device such as the electrooculogram (EOG) or eye tracking. In order to circumvent this problem, most validation and comparison studies focus on simulated data in which real or simulated ‘artifact-free’ data and ‘artifactual’ data are linearly mixed at some known ratio [11–21]. However, this approach is limited by the fact that simulated data do not entirely reflect true EEG characteristics with muscle contamination. For example, muscle activity does not always occur independently from the neural signals of interest, but simulations typically assume they do.

The few available validation studies which quantify artifact reduction performance on real data mostly focused on eye artifacts [22–24]. In order to compare BSS/ICA algorithms in more general settings, Delorme et al. [25] proposed measures to evaluate the quality of the obtained source components even when the ‘ground truth’ source components are unknown. They compared decompositions of 22 different BSS algorithms by evaluating measures of independence (based on mutual information) as well as the ‘dipolarity’ of the resulting source components. Here ‘dipolarity’ refers to the number of components whose scalp maps can be well explained by one equivalent dipole source. Such components are desirable since single independent sources, which the BBS/ICA algorithms aim to extract, would be represented by components of such characteristics. They find that mutual information-based ICA methods such as Infomax result in the highest number of near-dipolar components.

In this paper, our aim is to evaluate the quality of the cleaned data (in a sense the end product of the artifact reduction), which goes beyond the quality of the source signals (an intermediate product of the artifact reduction). A validation of the complete artifact reduction pipeline for muscle artifacts in real EEG data was carried out by McMenamin et al. [2]. However, different ICA algorithms were not compared.

To compare the overall muscle artifact reduction performance on real data, we here use a paradigm in which neural activity and muscle artifacts result in opposite effects: while muscle artifacts result in a broad increase in spectral power [1], the experimental paradigm induces neural activity that decreases spectral activity. More specifically, we use the well-known phenomenon of event-related desynchronization (ERD), that is, the decrease in oscillatory activity stimulus locked to an event. Eighteen participants were instructed to perform self-paced foot movements, which are known to be preceded by an ERD in the alpha band (8–13 Hz) and beta band (15–30 Hz) [26]. The recorded EEG signals also contain strong event-locked muscle artifacts as subjects moved their head rhythmically along with the foot movement. The average event-locked spectral activity then allows us to heuristically quantify the degree of muscle artifact contamination.

We compare the three most common ICA/BSS methods for EEG data, namely extended Infomax [27, 28], FastICA [29, 30] and SOBI/TDSEP [31, 32] with two linear decomposition methods that are not entirely ‘blind’ as they make use of the fact that the signals of interest are of oscillatory nature (Fourier-ICA [33] and spatio-spectral decomposition (SSD) [34]). To select the artifactual components, we use a previously validated automatic artifactual component classifier (IC_MARC, [35]).

The rest of the paper is organized as follows. We first describe the data set in Sect. 2.1, the compared methods in Sect. 2.2, the component classification in Sect. 2.3 and the methodology employed to evaluate overall artifact reduction performance in Sect. 2.4. The resulting ERDs and artifact contamination quantification are provided in Sect. 3 and finally discussed in Sect. 4.

## Methods

### Data

Data stem from a pre-measurement of a simulated driving experiment described in [36]. The experiment was conducted in accordance with the Declaration of Helsinki, and written informed consent was obtained from all participants. The self-paced right foot movements task was to press a brake pedal about once per second for five minutes. The electromyogram (EMG) was recorded with a bipolar montage at the knee of the right leg and the tibialis anterior muscle. Additionally, EEG was recorded from 64 approximately equidistant Ag/AgCl electrodes at 1000 Hz. For the presented offline analysis, EEG data were decimated to 200 Hz and broadband-filtered between 2 and 45 Hz (fifth-order Butterworth filter). Overly noisy electrodes were rejected using the variance criterion implemented in the function *reject_varEventsAndChannels.m* of the BBCI toolbox [37].

### Compared methods

We compare the ability of five linear decomposition methods to separate artifactual from neural activity. All five methods aim at solving the blind source separation (BSS) problem, in which the given EEG measurements $X \in \mathbb {R}^{M \times T}$ are modeled as being generated from the linear model $X = A S$. Here, *T* denotes the number of recorded data points, $S \in \mathbb {R}^{M \times T}$ denotes the time courses of *M* unknown sources, $A \in \mathbb {R}^{M \times M}$ denotes the unknown mixing process, and the number of electrodes is assumed to be equal to the number of source signals for simplicity. The goal is to recover the source signals *S* using very little information about the sources or the mixing process. Because this is an underdetermined problem, some assumptions have to be placed about the source signals to be recovered. A demixing matrix $\hat{W} \in \mathbb {R}^{M \times M}$ is estimated such that the estimated sources $\hat{S} = \hat{W} X$ best fulfill these pre-defined assumptions.

The overall aim of solving the BSS problem for artifact reduction is that artifactual and neural activities are separated into different source components. If this is the case, cleaner EEG signals can be reconstructed by discarding the artifactual source components.

#### ICA

Independent component analysis (ICA) solves the BSS problem using the assumption of mutually statistically independent sources. Several algorithms are available to solve this task, and we focus here on three of the most commonly used methods: extended Infomax [27, 28] as implemented in EEGLab [38], FastICA [29, 30] and SOBI/TDSEP [31, 32].

Extended Infomax and FastICA are classical ICA methods which use higher-order statistics to define independence. Infomax was derived from a neural network viewpoint, while FastICA maximizes the negentropy of the component distributions. Second-order methods make use of the temporal structure of the time series and require the recovered sources to be decorrelated over time. Here we use TDSEP (temporal decorrelation source separation) [32], which is equivalent to SOBI (second-order blind identification) [31]. TDSEP/SOBI aims to minimize the cross-covariances over several time lags between the estimated sources.

*Running ICA* We used extended Infomax, which finds both sub- and super-Gaussian sources, with the default settings in EEGLab for our analyses. We ran FastICA with the *symmetric* approach and all other options at default EEGLab values. We used code from A. Ziehe in the estimation of the TDSEP model, setting the number of time lags, $\tau$, to 99.

#### Fourier-ICA

Hyvärinen et al. [33] recently proposed to apply ICA on short-time Fourier transforms of EEG signals, in order to find more ‘interesting’ oscillatory sources than with time-domain ICA. The procedure optimizes the sparseness of the Fourier coefficients, which yields a separation of oscillatory signals at different frequencies.

Fourier-ICA has not been specifically designed to extract artifacts. In fact, the authors point out that time-domain ICA can be interpreted as maximizing non-Gaussianity. ICA may therefore be very well suited to find artifacts, which often are very non-Gaussian due to outliers in their time courses. Rather, the hope is that Fourier-ICA is better able to extract relevant oscillatory sources. In our setting, we aim to obtain clean oscillatory activity. Fourier-ICA might therefore be a promising method.

*Running Fourier-ICA* We used the implementation described in [33] to run Fourier-ICA with the default parameters. The minimum and maximum frequencies to be analyzed by Fourier-ICA were 15 and 30 Hz. We extracted as many components as there were channels.

#### SSD

Another recently proposed method for the extraction of oscillations is spatio-spectral decomposition (SSD) [34]. SSD aims to extract oscillations in a frequency band of interest at maximal signal-to-noise ratio (SNR). The goal is to maximize the signal power in the frequency band of interest while simultaneously minimizing it at the neighboring frequency bins. SSD extracts spatial filters $\mathbf{w} \in \mathbb {R}^M$ which maximize

1 $$\begin{aligned} \text {SNR}(\mathbf{w})&= \frac{\mathbf{w}^\top \Sigma _{\text {sig}} \mathbf{w}}{\mathbf{w}^\top \Sigma _{\text {noise}} \mathbf{w}} \end{aligned}$$

where $\Sigma _{\text {sig}}$ is the covariance of the data filtered in the frequency band of interest and $\Sigma _{\text {noise}}$ is the covariance of the data filtered in the sidebands. This problem reduces to a generalized eigenvalue problem and can be solved within a few seconds [34, 39]. SSD is a suitable preprocessing method for the analysis of neuronal oscillation [39–41]. Preliminary results for SSD on our data set were described in [42].

*Running SSD* We use 15–30 Hz as the frequency band of interest and 2 Hz long neighboring frequency bins. We extracted as many components as there were channels and ordered them according to their SNR.

#### High-pass filtering

It is well known that high-pass filtering EEG signals before applying ICA may improve the quality of the artifact separation [43, 44]. In fact, it is a fairly standard procedure to remove drifts prior to ICA-based artifact removal, and the benefit has been demonstrated in several studies [45–47]. Our data were already subjected to standard EEG processing, and on our band-pass-filtered data drifts are not a problem (cf. Sect. 2.1).

However, filtering at higher frequencies might also be beneficial when oscillatory processes are of interest. For example, trial-by-trial fluctuations of the blood-oxygen-level dependent (BOLD) signal were found to be positively correlated with high EEG gamma power when ICA demixing was obtained on gamma band-pass-filtered EEG data, but not when 30 Hz low-pass-filtered data were fed into ICA [48]. We might therefore benefit from a high cutoff frequency also in our study. Furthermore, we use information on the frequency band of interest for both Fourier-ICA and SSD. In order to obtain a fairer comparison to SSD and Fourier-ICA, we compute the demixing matrix for the three ICA methods both on the broadband-filtered data and on the data after a high-pass filter with a high cutoff frequency at 14 Hz (second-order Butterworth filter) had been applied.

To allow for a fair comparison of the broadband with the 14 Hz filtering condition, we proceed as proposed, e.g., in [47, 49]: for both filtering conditions, we apply the obtained demixing coefficients to the broadband-filtered data. In this way, we only consider the effect of filtering on the ICA decomposition, but not on the subsequent analysis.

### Automatic classification of estimated sources

Successful artifact removal relies on the correct identification of artifactual and non-artifactual components. This identification of artifactual component is a non-trivial task and requires time and expert knowledge. For a description of typical artifact components, we refer the reader to [50]. Here we use a previously validated automatic classifier of artifactual components, IC_MARC, to classify the sources estimated by each method [35]. IC_MARC was developed for sources derived by ICA, but may also be used to classify sources obtained from other methods.

IC_MARC assigns probabilities to independent components of belonging to each of six classes (blinks, lateral eye movements, electrical heart beat artifact, muscle artifact, neural or mixed artifact) and relies on multinomial regression to predict class probabilities for each component. We use these probabilities in two ways in this paper: (1) by classifying all components to the class for which the highest probability was predicted, we clean the data by removing all components not classified as neural and (2) we use the probabilities of the components being neural to determine the order of component removal. We use a version of IC_MARC which is based on a feature set containing only spatial features that we have seen to work well previously. IC_MARC tends to have a high specificity and sensitivity for the neural class with a balanced accuracy of 88% for 8023 independent components when training on one study and testing on another [35].

### Evaluation 1: event-related desynchronization (ERD)

We applied each method independently to the continuous EEG data and computed grand-average event-related (de-)synchronization (ERD/ERS) in the beta band (15–30 Hz), aligned to EMG peak activity.

ERD/ERS is calculated as the increase/decrease in signal power in a given frequency band relative to a reference period [51, 52]:

2 $$\begin{aligned} \text {ERD}(t)&:= \frac{\text {Power}(t) - \text {Reference \,power}}{\text {Reference \,power}} \end{aligned}$$

where $\text {Power}(t)$ denotes the average power over all trials at time point *t*. Here, we computed the time-resolved power by first band-pass filtering the signal in the beta band (15–30 Hz, fifth-order Butterworth filter), followed by computing its envelope using the Hilbert transform, and applying a moving-average over 100 ms. Epochs were aligned to EMG peak activity. These peaks were extracted from the rectified EMG as the maximum values within sliding windows of length [$-$ 750 750 ms] which exceeded a subject-specific threshold set by visual inspection. Our reference period was set to [$-$ 1200 $-$ 800 ms] before EMG peak activity.

**Fig. 1:**
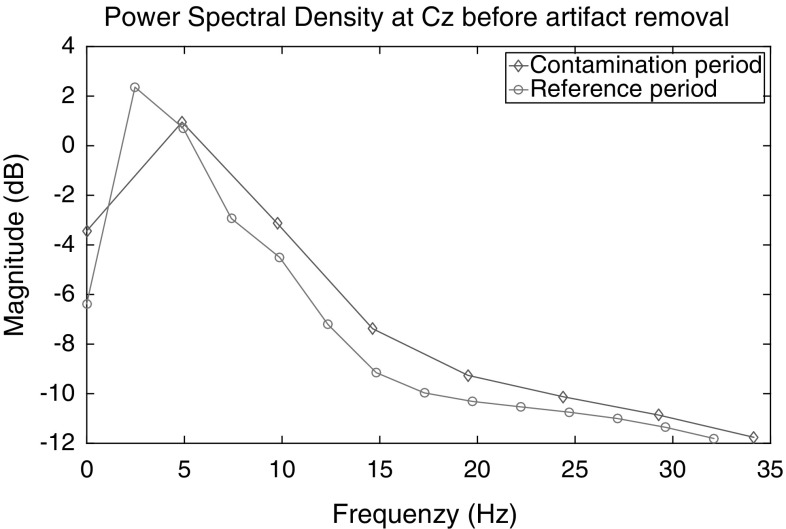
Grand-average power spectral density for uncleaned data during the artifact contamination [$-$ 100 100 ms] compared to the reference period [$-$ 1200 $-$ 800 ms]

**Fig. 2:**
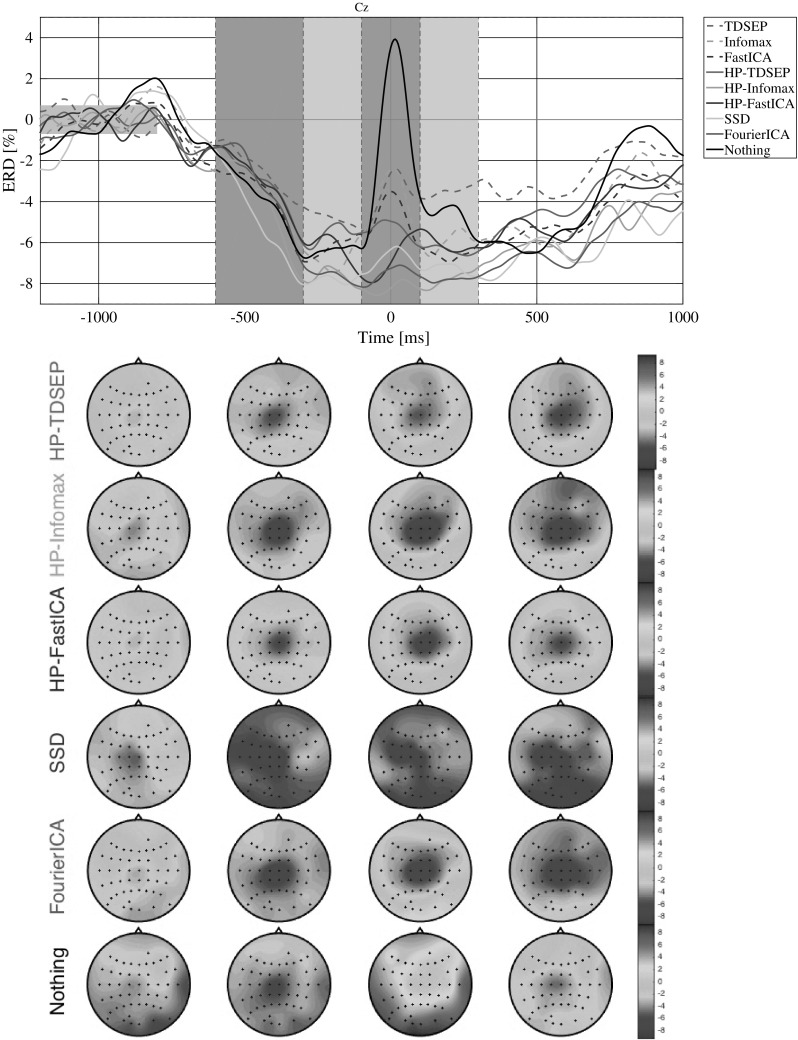
Grand-average event-related desynchronization (ERD) in the beta band (15–30 Hz), using EMG as a trigger. (Top) time courses of data reconstructed from neural ICs (and for SSD with the five components with highest SNR) at electrode Cz. (Bottom) ERD maps in the marked intervals ([$-$ 600 $-$ 300], [$-$ 300 $-$ 100], [$-$ 100 100], [100 300]) for selected methods

Voluntary movements are well known to elicit ERD in both the alpha and beta bands, most prominently over central sensorimotor areas and starting prior to movement onset (cf. [26]). The data additionally contain event-locked contamination in the form of a swift, strong peak at movement onset in the ERD of the beta band (cf. Fig. 2), which is probably due to subjects moving their heads along with the fairly rhythmical foot movement once per second. Contamination is strongest in the interval [$-$ 100 100 ms] around EMG peak activity. As shown in Fig. 1, it is characterized by a comparatively high power in higher frequencies, as expected for muscle artifacts.

The goal of artifact removal is to remove these muscle artifacts while retaining the neural activity. Here we can evaluate this goal because muscle and neural activities affect spectral activity in opposing direction: while muscle artifacts result in an increase in spectral power [1], the well-established neural signature of the task is a decrease in spectral power, i.e., ERD [26]. A signal which exhibits low ERD throughout the foot movement thus indicates, at the same time, the presence of the neural activity and a diminished influence of muscle artifacts.

Hence we aim to obtain a cleaner signal which exhibits low ERD. To quantify how well each method obtains this goal, we define the following heuristic ERD contamination measure as the peak ERD value during movement

3 $$\begin{aligned} \text {Peak\,(ERD)} := \max _{t \in [-100, \, 100]} \{\text {ERD}(t)\} \,, \end{aligned}$$

which we compute separately for each subject and preprocessing method. Here $\max \{\text {ERD}(t)\}$ is computed as the maximum value of ERD on the cleaned data between $-$ 100 and 100 ms relative to EMG peak activity.

In the presented data, the peak ERD score is positive when no artifact removal is carried out, as the muscle artifacts dominate. A preprocessing method which removes both artifacts and neural activity would result in a score of 0. An effective artifact removal method will reduce the artifacts, but keep the neural activity, and thus reduce the ERD to be highly negative throughout the foot movement. Therefore, the lower the peak ERD score, the better the artifact reduction method.

We use the ERD peak score to assess statistical significance when the cleaned data consist of only neural components. Furthermore, we evaluate the methods’ dependence on the number of source components retained. For each method, except SSD, we rank the obtained components by the probability of being an artifact as determined by IC_MARC. For SSD, we rank the components according to SNR. Retaining a smaller or larger number of sources corresponds to either a strict or soft policy for the removal of potential artifactual sources. Therefore, we vary the number of retained components from 1 to the number of channels, and we report the average ERD peak score over subjects.

### Evaluation 2: dipolarity

For comparison, we also compute a measure that does not depend on the classification of artifactual components or subsequent EEG analysis: the dipolarity measure proposed by Delorme et al. [25]. It is defined as the percentage of components whose scalp maps can be explained by one equivalent dipole source with less than a certain error variance. We use the EEGLAB implementation provided by Delorme et al. [25] and an error variance of 10%.

This dipolarity score is a simplistic, but very informative measure of physiological plausibility of the obtained ICA sources (see Delorme et al. [25] for a detailed discussion). In contrast, the ERD peak score measures the quality of the cleaned EEG signals in the beta band, which is, in a sense, the end product of the artifact reduction.

## Results

Figure 2 shows the grand-average ERD data with no cleaning and the same data cleaned by removing all non-neural sources for each method, except SSD for which we retained the five components with highest SNR. Results from applying the ICA methods with high-pass filtering are referred to with the prefix ‘HP’ (i.e., ‘HP-Infomax,’ ‘HP-FastICA’ and ‘HP-TDSEP’). The top of each figure contains the ERD time course at channel Cz, while the scalp maps corresponding to the intervals marked in light and dark gray are depicted for some of the best performing methods in the bottom part. As expected, we see a characteristical foot ERD over central sensorimotor areas before the foot movement. During the movement, we see the contamination of a time-locked muscle artifact across the whole scalp. The compared methods are able to reduce this artifact to varying degrees.

All three ICA methods improve if data are high-pass-filtered at a high cutoff frequency of 14 Hz before being decomposed. The lowest band power during the movement artifact is achieved by high-pass-filtered Infomax, followed by Fourier-ICA, SSD, high-pass-filtered FastICA and high-pass-filtered TDSEP. The ICA methods without the high-pass filtering perform the worst, but are nonetheless able to considerably reduce the artifacts. High-pass-filtered Infomax almost completely removed the artifact while maintaining the ERD.

**Table 1:** Average ERD peak score (lower is better) and dipolarity score (higher is better) with within subject error bars (cf. [53])

	ERD peak	Dipolarity
Nothing	$\,5.70 \pm 2.13$	
TDSEP	$-\,0.50 \pm 1.57$	$5.50 \pm 0.44$
Infomax	$-\,0.28 \pm 1.42$	$\mathbf {7}.\mathbf {00} \pm 0.49$
FastICA	$-\,1.44 \pm 1.14$	$5.00 \pm 0.37$
HP-TDSEP	$-\,1.64 \pm 1.34$	$3.17 \pm 0.34$
HP-Infomax	$-\,\mathbf {5}.\mathbf {46} \pm 1.07$	$6.44 \pm 0.56$
HP-FastICA	$-\,4.02 \pm 1.23$	$4.50 \pm 0.37$
SSD	$-\,3.98 \pm 2.28$	$1.56 \pm 0.48$
Fourier-ICA	$-\,4.47 \pm 1.11$	$4.89 \pm 0.33$

The best-performing method is highlighted in bold

The average ERD peak scores and dipolarity scores per method are shown in Table 1. For statistical testing on the ERD peak score, we specified two linear mixed models with the ERD peak score as dependent variable. The models were estimated using restricted maximum likelihood (REML) as implemented in the MATLAB Statistics Toolbox. As random effects, both models had intercepts for subjects and by-subjects slopes for each fixed factor in the model. First, to confirm the positive effect of high-pass filtering for ICA methods, we ran one model with the fixed factors ‘method’ (extended Infomax, TDSEP and FastICA) and ‘high-pass’ (yes or no) and their interaction. As expected, we found a significant positive effect of high-pass filtering ($F(1, 102) = 9.2, p < 0.01$). In the second model, we included all five linear decomposition methods in their best variants (i.e., the high-pass versions if applicable) as well as the nothing condition. The method had a significant effect ($F(5, 102) = 4.5, p < 0.01$). Post hoc pairwise comparisons between the methods showed that all five decomposition methods significantly improved over no artifact reduction (all $p < 0.01$), and HP-Infomax improved significantly over HP-TDSEP ($p < 0.05$).

For statistical testing on the dipolarity score, we specified the same two linear mixed models, but with the dipolarity score as dependent variable. In contrast to the average ERD peak score, high-pass filtering had a significant negative effect on the dipolarity score ($F(1, 102) = 9.1, p < 0.01$). The second model compared the five decomposition methods HP-TDSEP, HP-Infomax, HP-FastICA, SSD and Fourier-ICA and found a significant effect of the method ($F(4, 85) = 13.1, p < 0.001$). Post hoc pairwise comparisons between the methods found almost all compared methods to be significantly different from each other, with SSD being significantly worse, and HP-Infomax being significantly better than the other four methods ($p<0.05$).

Let us note that all investigated artifact removal methods remove variance from the signals and thus reduce the power in all frequencies. They do so with varying degrees. The average reference power entering ERD computation in Eq. (2) was reduced strongest by SSD, followed by HP-Infomax, HP-FastICA, HP-TDSEP, Fourier-ICA, FastICA, Infomax and TDSEP.

**Fig. 3:**
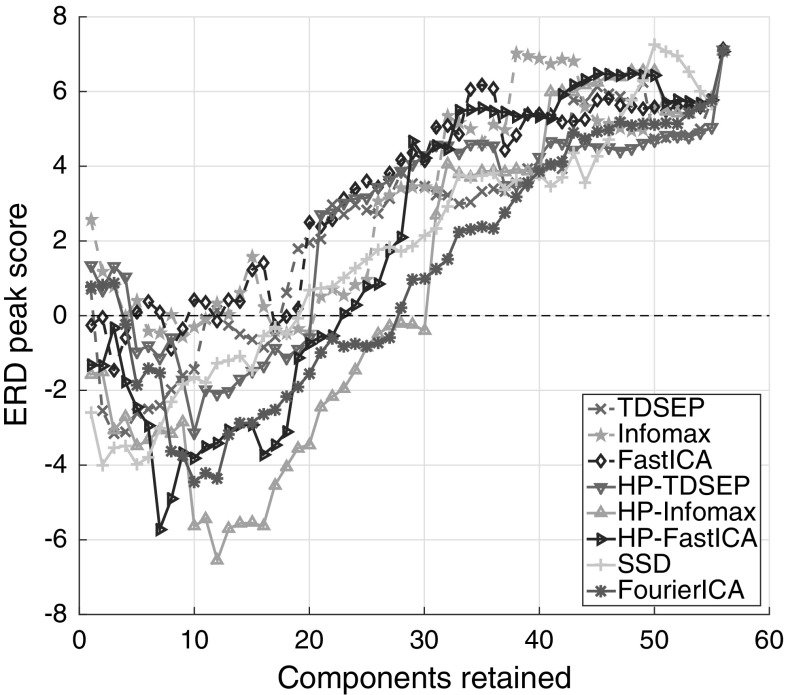
ERD peak score measure in dependence of the number of components retained. Lower is better. A method which removes all neural and muscle activity would be at 0 (dashed black line)

The retained variance per method can be influenced by changing the number of components that is removed. Figure 3 shows the ERD peak score as a function of the number of components retained. Components were removed in order of decreasing probability of being neural as determined by IC_MARC, except for SSD, where components were removed in order of decreasing SNR.^1^ The results are in line with the general picture presented in Fig. 2 and Table 1, which show the case of retaining all ICs whose highest probability was for the neural class. High-pass filtering the data at a high cutoff frequency of 14 Hz tends to improve the ERD peak for the ICA methods. High-pass-filtered extended Infomax obtains the best (lowest) ERD peak score over all numbers of components retained and retains its leading position over a wide range of number of components retained (10–25 components).

## Discussion

In this paper, we analyzed and compared the artifact reduction capabilities of the three most common time-domain ICA methods (extended Infomax, FastICA and TDSEP) with two other linear decomposition methods which are tailored to extract oscillatory signals (Fourier-ICA and SSD). As ICA and other linear decomposition methods are the most widely used tool to reduce muscle artifacts in EEG signals, many researchers wonder which one to choose in practice. However, it remains difficult to compare different artifact reduction algorithms on real data as the ‘ground truth’ artifact-free signals are unknown. Here we resorted to a self-paced movement paradigm which induces a decrease in rhythmic activity, as opposed to muscle artifacts which typically increase spectral power. While our study is also limited by the lack of a firm ground truth of the underlying neural activity, it allowed us to heuristically evaluate the ability of the compared methods to remove a strong event-locked muscle artifact while maintaining neural activity in the form of event-related desynchronization. Our findings indicate that all five methods were able to remove much of the movement artifact, with extended Infomax—after adequate high-pass filtering—performing best.

We also evaluated the methods’ dependence on the number of source components retained. It is reassuring that the performances of the methods, relative to each other, remain at a similar level for a wide range of numbers of components retained. This indicates that there are indeed true differences between the methods that do not strongly depend on whether a strict or mild cleaning policy is used. High-pass-filtered Infomax yielded the best ERD peak score over a range of retained components.

Consistent with Delorme et al. [25], we find that extended Infomax performs best, both in terms of the dipolarity score proposed in Delorme et al. [25] and in terms of our ERD peak score. However, the ERD peak score, which heuristically quantifies an oscillatory phenomenon in the cleaned data, is not as sensitive as the dipolarity measure, which is computed on the source signals. This is to be expected, since the source signals are an intermediate step within the artifact reduction, and differences on this intermediate level may not necessarily translate into strong differences in the cleaned data. Indeed, observed ERD differences between the methods are rather small, which suggests that the choice of the decomposition method may often not result in strong differences in data quality.

Our results indicate that adequate high-pass filtering may be more important than the choice of the ICA method: all three ICA methods achieved a better ERD peak score when the data had been high-pass-filtered at the cutoff frequency just below the frequency band of interest before decomposition. The effect was most prominent for Infomax and FastICA. However, high-pass filtering at the cutoff frequency had a negative impact on the dipolarity score. The effect of high-pass filtering thus strongly depends on the intended subsequent analysis and is not always beneficial. Filtering might guide the decomposition toward extracting the components that explain the activity we are interested in. That is, if (and only if) we are not interested in low frequencies in further analysis, we may benefit from removing them before ICA decomposition. This effect seems to be relevant, probably because the low-frequency parts of an EEG signal contain a large portion of its variance.

Compared with high-pass-filtered Infomax, both SSD and Fourier-ICA achieve only slightly inferior, still impressive performance in terms of the ERD peak scores. SSD yields the lowest number of interpretable dipolar components, but seems particularly suited to determine only a few number of neural components. Because SSD is faster to evaluate, it may be a good compromise between the time it takes to decompose the data and the quality of artifact separation.

Our results indicate that ICA and other decomposition methods were suitable tools to remove muscle artifacts from our EEG data. This is especially interesting since the observed muscle artifacts do not occur independently from motor planning neural activity—which clearly violates ICA’s assumptions. A co-activation of artifacts and neural activity is quite common in practice. Our results complement the findings from McMenamin et. al [2], which suggest that ICA is still a sensible choice even in those settings.

## Footnotes

1 We also looked at the results for SSD with components chosen according to IC_MARC, as for the other methods. However, the performance resulting from this component selection was lower than that using the SNR. For SSD, we therefore only present the results using SNR.
